# Numerical study on the atomization performance of aviation biofuel with high blending ratio

**DOI:** 10.1371/journal.pone.0321880

**Published:** 2025-05-06

**Authors:** Yanyu Cui, Changhong Xiong, Shugang Yang, Qingmiao Ding, Kai Zhang, Chen Xu

**Affiliations:** 1 Civil Aviation University of China, Dongli District, Tianjin, China; 2 Liaohe Oilfield of China National Petroleum Corp, Panjin, Liaoning, China; 3 Tianjin Jeppesen International Flight College, Tianjin, China; NED University of Engineering and Technology, PAKISTAN

## Abstract

Blending aviation biofuel with conventional jet fuel helps reduce carbon emissions in aviation. However, high blending ratios (≥50%) can impact engine atomization performance. This study uses a three-dimensional simulation of gas-liquid flow in a centrifugal nozzle with the volume of fluid (VOF) method to evaluate the atomization performance of aviation biofuels with traditional jet fuel at different blending ratios (0%, 40%, 60%, 80%, 100%), under varying temperatures and nozzle inlet pressures. The simulation results align well with empirical data, showing deviations of less than 10%. As the inlet pressure increases from 0.2 MPa to 1.0 MPa, the nozzle outlet velocity increases by 127.0%, and the liquid film thickness decreases by 41.8%, improving atomization performance. At 0.2 MPa, higher blending ratios (≥50%) leads to thicker liquid films, reducing atomization efficiency. However, at 1.0 MPa, the effect of blending ratio on atomization becomes less significant, with the difference in liquid film thickness reduced to 4.2%. Fuel temperature significantly affects atomization, with more noticeable differences between low- and high-blending fuels. As temperature rises from 0°C to 50°C, liquid film thickness decreases, with a reduction of 14.6% for low-blending fuel and 52.8% for high-blending fuel.

## 1. Introduction

Aviation biofuels are a crucial pathway for achieving low-carbon development in the aviation industry due to their reduced carbon footprint and substantial potential to lower greenhouse gas emissions. As a carbon-neutral alternative, aviation biofuels blended with conventional jet fuels can significantly reduce CO₂ emissions. Currently, approved aviation biofuels support blending ratios of up to 50% (by mass), which can result in CO₂ emission reductions of up to 40% [1]. Increasing the blending ratio offers significant opportunities for further mitigating CO₂ emissions.

In aviation engines, fuel atomization is essential for enhancing heat and mass transfer within the combustion chamber, which in turn improves combustion efficiency [2]. In contrast, poor atomization can impair ignition and increase emissions [3]. The blending ratios of aviation biofuels significantly affect various engine performance parameters, with atomization performance being a key factor. As a result, many researchers have conducted extensive studies on the atomization characteristics of aviation biofuels.

Sivakumar [4], Sakthikumar [5], and Vankeswaram [6] investigated the atomization performance of pure Jatropha-derived hydro-processed renewable jet (HRJ) fuel, pure Jet A-1, and a 20% HRJ blend with Jet A-1 using a single swirl nozzle, an aviation engine nozzle, and a hybrid air atomizer (HAA), respectively. These studies revealed that, at inlet pressures between 0.2 and 0.4 MPa, the atomization performance of pure HRJ and low-blend HRJ fuels was slightly lower than that of Jet A-1. However, while these studies explored various nozzle types, they did not assess atomization performance across a broader pressure range or investigate high-blend aviation biofuels, which often exhibit larger liquid film thicknesses, lower exit velocities, and larger Sauter mean diameter (SMD) due to their different physical properties, such as higher viscosity and surface tension.

In addition, Zhao [7] and Zhou [8] examined the atomization performance of pure RP-3 and Fischer-Tropsch (FT) fuels at pressures ranging from 0.1 to 1.0 MPa. Their findings indicated that FT fuel exhibited a wider spray cone angle, suggesting distinct atomization behaviors compared to conventional RP-3 fuel. These studies primarily focused on pure conventional fuels and pure biofuels separately and did not explore the behavior of blends, which have more variable physical properties.

Furthermore, Kumaran Kannaiyan [9] compared the spray characteristics of synthetic gas-to-liquids (GTL) fuel and Jet A-1, reporting that GTL fuel demonstrated faster droplet breakup and dispersion at higher pressures due to its lower viscosity and surface tension. Although both fuels displayed similar overall spray characteristics, the differences in their physical and chemical properties significantly influenced local spray behavior, highlighting the importance of viscosity and surface tension in atomization. These findings further reinforce the need to understand how biofuels, with their unique chemical properties, such as higher viscosity and surface tension, behave under varying conditions.

Ding et al. [10] showed that increasing the biofuel blending ratio in conventional jet fuel raised viscosity, which negatively impacted atomization performance by increasing the SMD. This, consequently, led to reduced combustion efficiency and higher pollutant emissions [11–15]. These studies have explored various factors such as nozzle type, fuel type, and pressure, but their investigations are not comprehensive. The pressure range examined is relatively narrow, and the blending ratios considered are limited, mostly focusing on low-blend biofuels. As a result, the effect of viscosity changes due to higher biofuel blending ratios on atomization performance has not been adequately addressed. Therefore, the impact of viscosity changes on atomization performance, which is closely tied to the blending ratio, remains critical and warrants further investigation. This gap in the existing literature serves as a key motivation for the present study, which aims to examine high-blend biofuels and their atomization behavior across a broader range of pressures, blending ratios.

During gas turbine operation, fluctuations in external environmental temperatures, particularly at varying flight altitudes and speeds — can significantly affect fuel temperature. Additionally, heat transfer from lubricating oil, compressed air, and combustion flames can further influence fuel temperature [16]. Changes in fuel temperature alter its physical properties, such as density, dynamic viscosity, and surface tension, which directly impact atomization characteristics. Renata and Lefebvre [17,18] found that increasing diesel temperature reduced the average droplet size and broadened the droplet size distribution. Similarly, Liu et al. [16] reported that higher temperatures for conventional jet fuel resulted in a wider spray angle and thinner liquid films. While these studies emphasize the importance of temperature effects on the atomization of conventional fuels, limited research has explored the atomization characteristics of aviation biofuels under varying temperature conditions.

Numerical simulation has become an essential tool for studying the fuel atomization process, especially in analyzing complex flows and spray behaviors. Among these numerical methods, the VOF method is widely applied in fuel atomization simulations. The VOF method accurately models the formation and development of sprays by tracking the interface between the liquid and gas phases. As a result, the VOF method has become one of the most used numerical tools in spray simulation. For instance, Pierre et al. [19], Qiao at al. [20], Chen et al. [21], Zhang et al. [22] and Yu et al. [23] have all used the VOF method to simulate fuel atomization, validating its effectiveness and accuracy in atomization studies. These studies showed that the VOF method provided reliable numerical predictions for fuel atomization processes, especially in complex spray environments, highlighting its promising application potential.

In summary, while existing studies provide valuable insights into the atomization performance of low-blend or pure biofuels, they predominantly focus on lower blending ratios. The atomization characteristics of high-blend biofuels (with biofuel content exceeding 50%) remain underexplored. This gap arises because higher biofuel blends can induce more complex physical phenomena in the atomization process, such as changes in liquid film thickness and spray cone angle, which significantly affect combustion efficiency and emission characteristics. Therefore, the motivation of this study is to address this gap by conducting an in-depth investigation of the atomization characteristics of high-blend biofuels.

To tackle this issue, this study employs the VOF method to simulate the atomization process of aviation biofuels in a simple centrifugal nozzle. The contribution of this research lies in systematically exploring the effects of blending ratio, inlet pressure, and fuel temperature on primary atomization characteristics, providing quantitative data on key parameters such as liquid film thickness, spray cone angle, and outlet velocity. These data not only enhance the theoretical understanding of high-blend biofuel atomization but also provide crucial support for the wider adoption of aviation biofuels.

## 2. Model construction and numerical methods

### 2.1 Geometric model and mesh generation

**Fig 1:**
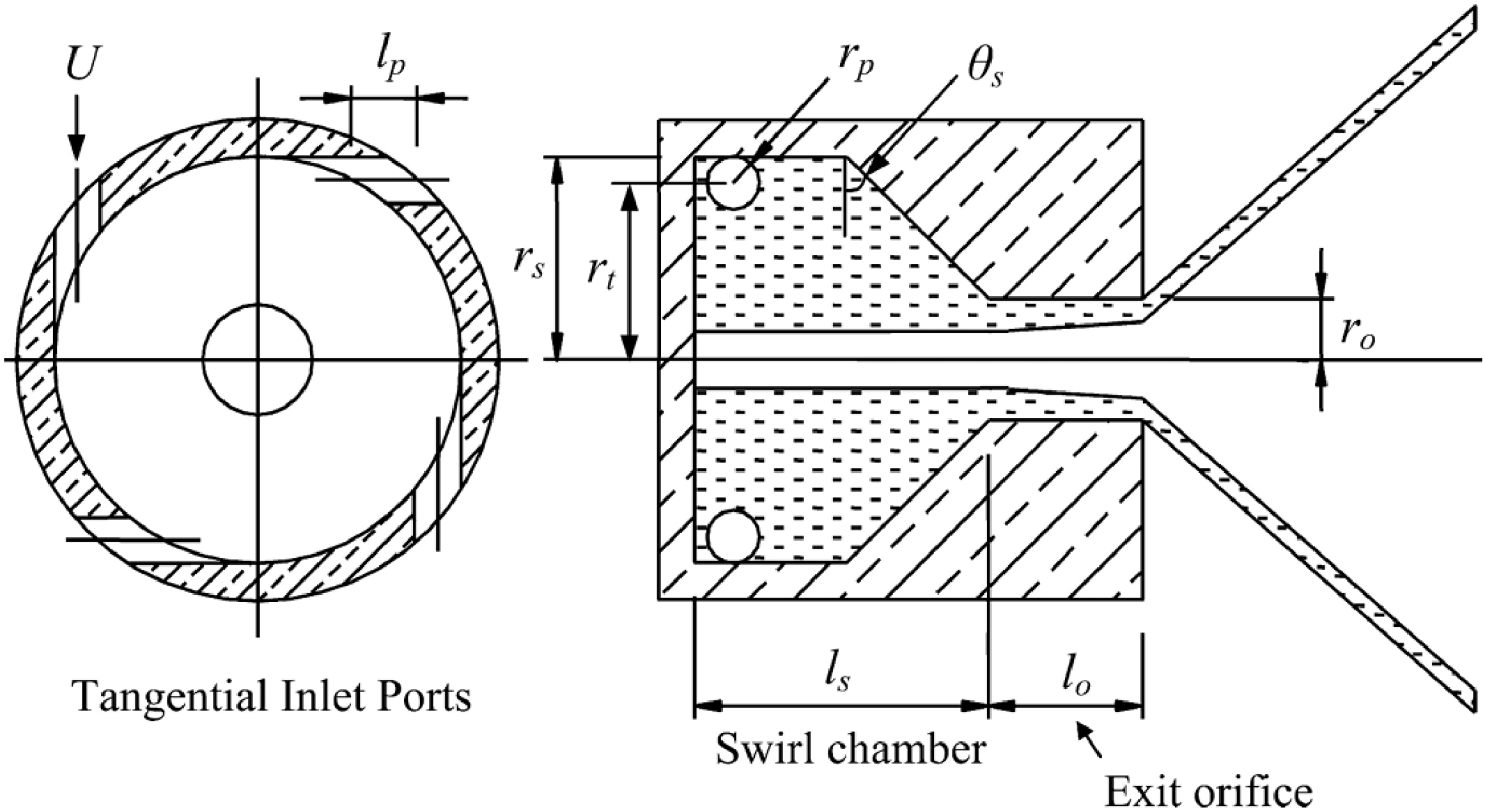
Typical structure of a centrifugal nozzle [25].

In aviation engines, centrifugal nozzles are among the most used atomizers and play a critical role in fuel atomization. They are often employed as the primary atomization components in combination-type atomizers [24–26]. A typical centrifugal nozzle consists of three main parts: the inlet, swirl chamber, and outlet, as shown in Fig 1 This design efficiently converts pressure energy into kinetic energy, generating a high relative velocity between the ejected liquid and the surrounding gas [27]. The operating principle involves liquid entering the swirl chamber through tangential ports, which imparts a high angular velocity to the flow. This swirling motion forms a vortex with a gas core at the center of the swirl chamber [28]. The liquid is then discharged through the nozzle, producing a hollow-cone spray.

The modeling in this study strictly follows the design guidelines for centrifugal nozzles as outlined in the Aviation Engine Combustion Chamber Design Manual. These guidelines include specifications for the length of the swirl chamber $l_{s}\approx 2.2r_{s}$ , the convergence angle of the nozzle inlet $\alpha =90^{{}^{\circ}}$, and the length of the nozzle outlet $0.15r_{o}\leq l_{o}\leq 2r_{o}$ [29]. The specific parameters are presented in Table 1.

**Table 1:** Geometric parameters of the centrifugal nozzle.

Structure parameters	Value
Orifice diameter	3.0 mm
Orifice length	0.6 mm
Swirl chamber diameter	10.0 mm
Swirl chamber height	4.6 mm
Inlet diameter	1.5 mm
Number of swirl slot	4

**Fig 2:**
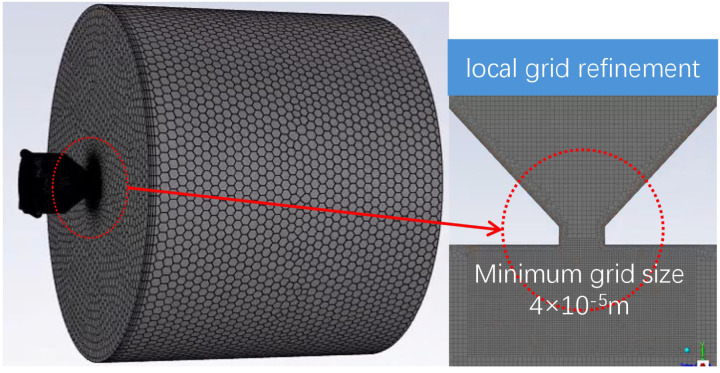
Mesh generation details.

Hexahedral meshes are employed for domain discretization in Fluent. To minimize boundary effects in the outlet region, a cylindrical extension with a diameter and length 20 times that of the nozzle is added to the nozzle outlet. The Fluent simulation focuses primarily on the internal flow within the nozzle and the detailed flow characteristics near the outlet region. Consequently, mesh refinement is applied inside the nozzle and in the outlet area, as shown in Fig 2. The mesh size is set to 4 × 10^−5^ m. All preprocessing and simulations are performed using ANSYS Fluent 2022 R1.

**Fig 3:**
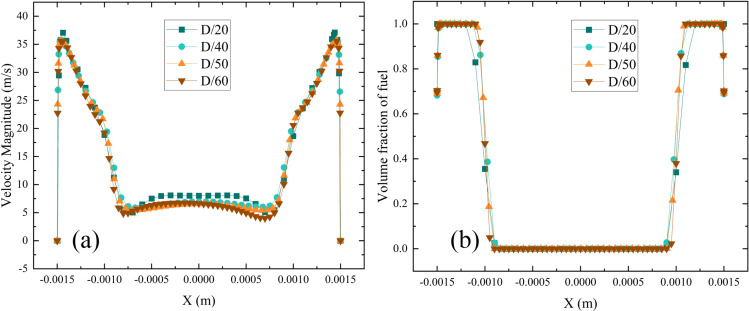
Mesh independence verification. (a) Velocity magnitude variation curves with mesh densities, (b) Fuel volume fraction variation curves with mesh densities.

To ensure mesh independence, various mesh densities (D/20, D/40, D/50, D/60), where D denotes the outlet diameter, are tested. As shown in Fig 3, once the mesh density reaches D/50, further refinement has no significant impact on the liquid volume fraction or outlet velocity. Therefore, balancing computational efficiency and accuracy, a mesh density of D/50 is selected as the optimal choice.

### 2.2 Governing equations and numerical methods

The fuel atomization process is a typical two-phase flow problem, simulated using the VOF method, which solves the continuity equation. The VOF method tracks the volume fraction of each phase within a control volume to determine the phase distribution. The sum of the volume fractions for all phases within a single control volume equals 1. In this simulation, air represents the gas phase, while the fuel represents the liquid phase.


$$\frac{\partial (\alpha_{j}\rho_{j})}{\partial t}+\frac{\partial (\alpha_{j}\rho_{j}u_{j})}{\partial t}=0$$ (1)

In Equation (1), $\alpha_{j}$, $\rho_{j}$, $u_{j}$ represent the volume fraction, density, and velocity of the $j_{th}$ phase, respectively. The density *ρ* and viscosity *μ* within a control volume are calculated using Equations (2) and (3).


$$\rho (\alpha )=\alpha \rho_{l}+(1-\alpha )\rho_{g}$$ (2)

$$\mu (\alpha )=\alpha \mu_{l}+(1-\alpha )\mu_{g}$$ (3)

In gas-liquid two-phase flow, the VOF method introduces the volume fraction *α* of the fluid in each computational cell. When α=1, the cell is fully occupied by liquid. When α=0, the cell is fully occupied by gas. Consequently, a volume fraction between 0 and 1 indicates that the cell represents the gas-liquid interface. $\rho_{l}$ and $\rho_{g}$ denote the densities of the liquid and gas, respectively, while $\mu_{l}$ and $\mu_{g}$ represent the viscosity of the liquid and gas, respectively.

The Realizable k–*ε* turbulence model is employed, and the remaining solver settings are provided in Table 2.

**Table 2:** Summary of solver settings.

Numerical Scheme	Solver Settings
VOF model	Explicit; Geo-Reconstruct
Pressure-Velocity coupling	SIMPLEC
Pressure discretization method	PRESTO!
Momentum discretization method	2nd Order Upwind
turbulence model	Realizable k– *ε*

### 2.3 Data processing methods and simulation validation

**Fig 4:**
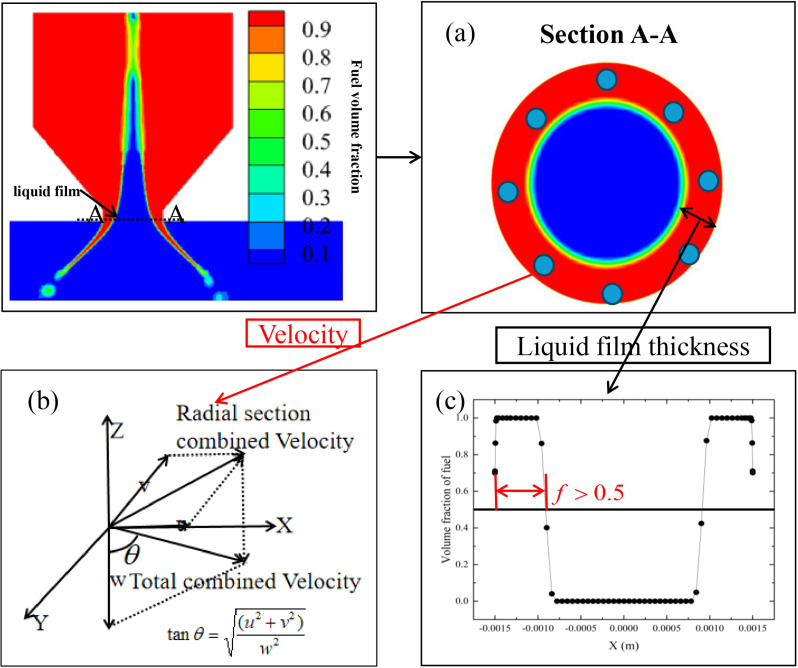
Data processing methods. (a) Liquid phase distribution at the outlet interface, (b) Spray half-cone angle, (c) Fuel volume fraction distribution.

As shown in Fig 4, during data processing, the velocity in each direction at the outlet is averaged using data from eight evenly distributed points where the liquid volume fraction at the nozzle outlet is 1. The spray half-cone angle is calculated based on the directional velocity (u,v,w). The liquid film thickness is determined by defining the region where the volume fraction *f* > 0.5 as the liquid film area. The distance from the point where *f* = 0.5 to the wall is taken as the liquid film thickness. The applicability of this data processing method has been validated in references [30,31].

**Fig 5:**
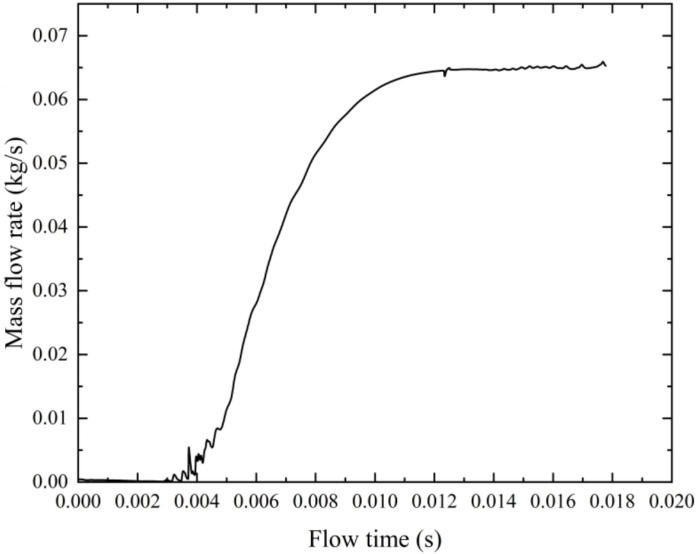
Variation of nozzle outlet mass flow rate with flow time for steady-state confirmation.

The convergence criterion for the simulations is set based on the residuals of the governing equations, including continuity, momentum (in the *x*, *y*, and *z* directions), as well as turbulence quantities k and *ε*. The solution is considered converged when the residuals of these equations drop below a threshold of 1×10⁻⁶. Additionally, convergence is further confirmed by monitoring key physical quantities to ensure consistency and accuracy across iterations. As shown in Fig 5, the nozzle outlet flow rate is used as the stability criterion to confirm that the simulation has reached a steady state. The simulation is considered to have reached a steady state when the outlet mass flow rate becomes stable.

To verify the accuracy of the simulation, the results are compared with the equation proposed by Rick and Lefebvre [32], which is based on a large set of experimental data fitted to predict liquid film thickness and axial velocity. However, special attention must be given to the assumptions and limitations of this empirical formula, particularly when applied to high-blending-ratio fuels, as these assumptions may affect the accuracy of the validation process. The following is a detailed discussion of these assumptions and limitations.

Assumptions of the Empirical Formula:

The equation by Rick and Lefebvre is derived from standard liquid fuels (such as water or diesel) under typical conditions, with the following basic assumptions:

(1) Stable Fluid Properties: The formula assumes that the fluid used has stable physical properties, particularly viscosity and density, which remain constant throughout the experimental process. This allows the equation to accurately describe the liquid film thickness and axial velocity.
(2) Spray Nozzle Geometry Assumption: The equation assumes that the nozzle geometry remains consistent, specifically the outlet diameter and swirl chamber length, as variations in nozzle design can significantly influence the flow behavior. In the experimental setup of Rick and Lefebvre, different nozzle geometries were tested with outlet diameters of 1.2 mm and 2.4 mm, inlet diameters of 0.8 mm and 1.1 mm, and swirl chamber diameters of 8 mm and 10 mm. The nozzle geometry used in this study closely matches that of the experimental setup, ensuring the applicability of the formula.
(3) Experimental Pressure Range Assumption: The equation is based on experiments conducted within a pressure range of 0.5 to 2.7 MPa. Therefore, the applicability of the equation is more accurate within this range, and deviations may occur at very low or very high pressures.

Limitations for High-Blending-Ratio Fuels:

High-blending-ratio fuels usually exhibit higher viscosity, which may cause the liquid film thickness to deviate from the predictions of the empirical formula, potentially affecting atomization and injection performance. When these altered properties differ substantially from the conditions under which the empirical formula is originally developed, the predictive accuracy can diminish. Consequently, designers and researchers must exercise caution in applying such formulas to high-blending-ratio fuels and consider re-calibrating or refining the correlations to account for the unique physicochemical characteristics of these fuels.

The empirical formula is given as follows:


$$t=3.66{\left(\frac{md_{0}\mu_{f}}{\rho_{f}\Delta p}\right)}^{0.25}$$ (4)

$$U_{a}=\frac{m}{\rho_{f}\pi t(d_{0}-t)}$$ (5)

In the equation, *t* represents the liquid film thickness, $U_{a}$ is the axial velocity of the fuel at the outlet, *m* is the mass flow rate, $d_{0}$ is the outlet diameter, $\mu_{f}$ is the fuel viscosity, and $\rho_{f}$ is the fuel density.

The nozzle geometry in this study is closely aligned with the experimental setup, which ensures that the assumptions regarding nozzle design are valid. Although the comparison pressure range (0.2 to 1.0 MPa) does not exactly match the fitting range (0.5 to 2.7 MPa), the pressure ranges are sufficiently similar to permitting a valid comparison. Moreover, the fuel used here has comparable physical properties, such as viscosity and density, to those used in Rick and Lefebvre’s experiments. Given the alignment in nozzle design, pressure range, and fuel properties, the use of their empirical formula for simulation validation is justified.

**Fig 6:**
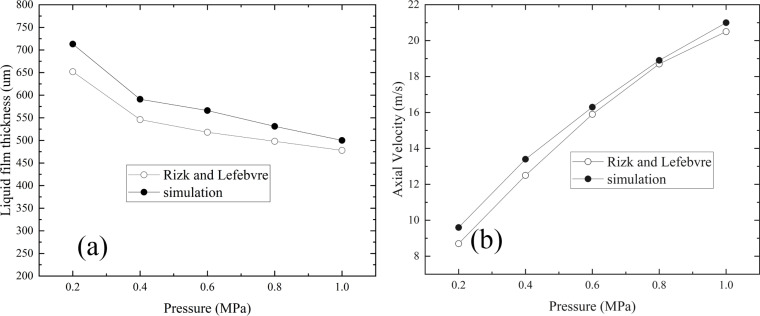
Comparison between simulation results and empirical formula results. (a) The variation of liquid film thickness with pressure, (b) The variation of axial velocity with pressure.

The simulation results from Fluent are compared with those predicted by Rick and Lefebvre’s formula (Fig 6). The comparison reveals that the Fluent simulation accurately captures the trends in liquid film thickness and axial velocity, with errors consistently within 10%. Thus, the simulation results validate the modeling approach and confirm the accuracy of the simulation process.

### 2.4 Simulation conditions

The simulation parameters are provided in Table 3. Fluent is used in this study to simulate the internal flow within the nozzle for various blending ratios of aviation biofuel (B0, B40, B60, B80, B100), where B0 and B100 represent unblended and 100% aviation biofuel, respectively, under different inlet pressures. The inlet pressures, ranging from 0.204 MPa to 1.034 MPa (0.2, 0.4, 0.6, 0.8, and 1.0 MPa), are selected based on typical fuel injection pressures used in aviation engines [33]. This pressure range enables the investigation of the effects of varying pressures on the primary atomization characteristics of aviation biofuels with different blending ratios. The key physical and chemical properties of aviation biofuels at different blending ratios [10] are presented in Table 4.

**Table 3:** Simulation parameters.

Category	Parameter	Category	Parameter
Pressure inlet	0.2-1.0MPa	Time step size	5 × 10^–6^ s
Total run time	until the flow rate stabilizes	Wall	No slip
Air density	1.225 kg/m^3^	Air Viscosity	1.7894 × 10^-5^N s/m^2^
Fuel type	B0, B40, B60, B80, B100		

**Table 4:** Key physical and chemical properties of aviation biofuels with different blending ratios.

Properties	B0	B40	B60	B80	B100
Density(20°C, kg/m^3^)	793.8	780.4	776.7	772.9	769.4
Kinematic Viscosity (20°C, mm^2^/s)	1.575	1.974	2.096	2.183	2.368
Surface Tension (mN/m)	24.6	24.7	24.9	25.0	25.1

## 3. Results and discussion

In studying the primary atomization characteristics of a centrifugal nozzle, three key parameters including liquid film thickness, outlet velocity, and spray cone angle directly influence atomization performance. Liquid film thickness determines the size distribution of spray droplets, especially in a centrifugal nozzle, where the thickness of the annular liquid film affects droplet formation and distribution [34]. Outlet velocity impacts the breakup of the liquid film and droplet formation by increasing the liquid’s kinetic energy and shear forces [35]. Additionally, the spray cone angle not only influences the distribution of droplets within the combustion chamber but also plays a crucial role in ignition performance, combustion efficiency, and pollutant emissions [36]. Therefore, a thorough analysis of these parameters is essential for evaluating the atomization characteristics of aviation biofuels with varying blending ratios.

### 3.1 Effect of nozzle inlet pressure on atomization performance

**Fig 7:**
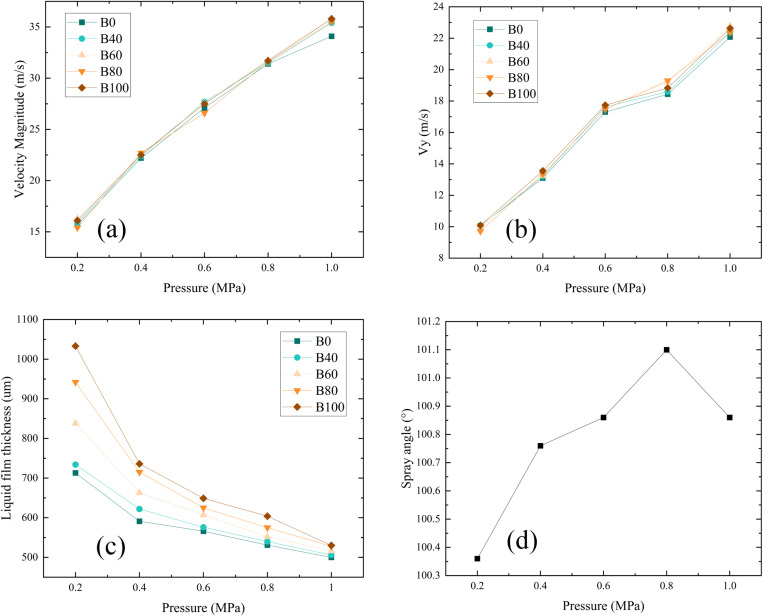
Variation of fuel atomization performance under different inlet pressures. (a) Outlet total velocity, (b) Axial velocity, (c) Liquid film thickness, (d) Spray cone angle.

Nozzle inlet pressure significantly influences fuel atomization performance. The variations in outlet velocity, liquid film thickness, and spray cone angle of aviation biofuel under different pressures are presented in Fig 7. As the inlet pressure increases from 0.2 to 1.0 MPa, the total outlet velocity rises by 127.0%, while the axial velocity increases by 119.4%. This indicates that higher inlet pressures substantially enhance the fuel’s kinetic energy, enabling it to be ejected at greater velocities, thereby improving atomization performance. Furthermore, as the pressure increases from 0.2 MPa to 1.0 MPa, the liquid film thickness decreases by 41.8%, suggesting that the increased shear forces at higher pressures facilitated faster breakup of the liquid film into droplets, further enhancing atomization quality. This behavior is consistent with the observations of Dong [37], who demonstrates that elevated pressures intensify the interaction between liquid and air phases, leading to reduced liquid film thickness, enhanced atomization efficiency, and increased fuel injection velocity.

However, changes in the spray cone angle from 0.2 to 1.0 MPa have no significant effect. This observation aligns with Taylor’s theory [38], which predicts that the spray cone angle is primarily determined by the geometry of the swirl chamber rather than the inlet pressure. In summary, increasing the nozzle inlet pressure significantly boosts outlet velocity and reduces liquid film thickness, thereby improving fuel atomization quality.

### 3.2 Effect of blending ratio on atomization performance

**Fig 8:**
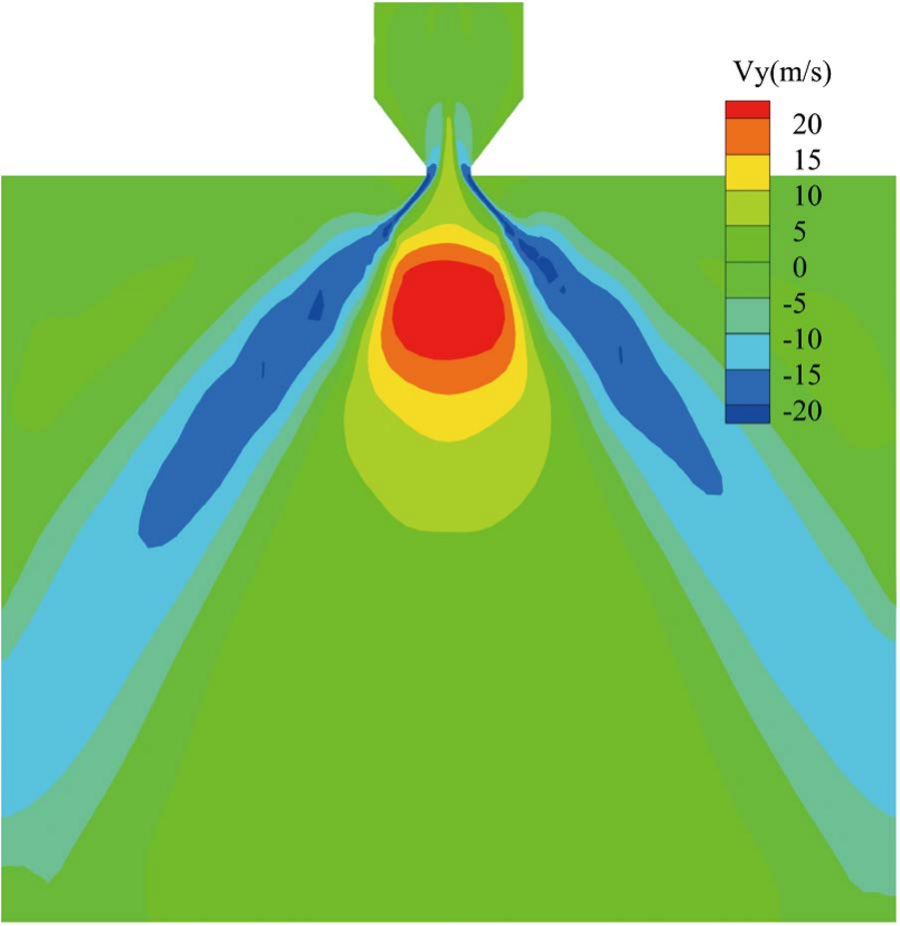
Cross-sectional velocity distribution contour.

Fig 8 presents the cross-sectional axial velocity distribution contour at a nozzle inlet pressure of 1.0 MPa and a fuel temperature of 25°C. It is evident that the axial velocity of the fuel ejected from the nozzle increases significantly. Near the wall, the axial velocity direction is opposite to that in the central region of the nozzle. This phenomenon is primarily due to the fact that air is drawn into the nozzle, resulting in the formation of an air-core structure. The velocity at the center of the nozzle reflects the air intake velocity, not the fuel flow rate.

**Fig 9:**
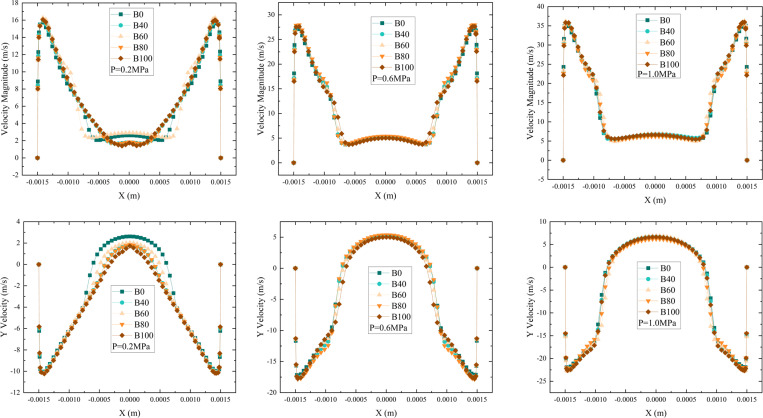
Variation of outlet velocity for aviation biofuels with different blending ratios at different pressures. (Left: *p* = 0.2 MPa, Middle: *p* = 0.6 MPa, Right: *p* = 1.0 MPa).

The velocity distribution at the outlet plane varies significantly with different blending ratios of aviation biofuels. As shown in Fig 9, at a pressure of 0.2 MPa, both the total outlet velocity and axial velocity exhibit noticeable differences in the flat central region. The velocity in this region reflects the air entrainment speed, indicating that as the blending ratio increases, the higher viscosity of the fuel reduces air entrainment in the central region. As the pressure increases to 0.6 MPa, these velocity differences gradually diminish. By the time the pressure reaches 1.0 MPa, no significant differences are observed in the total outlet velocity or axial velocity distribution among fuels with varying blending ratios.

At low pressure, the higher viscosity of the fuel restricts flow, weakens the air entrainment effect and reduces velocity in the central region. However, as pressure increases, kinetic energy and shear forces within the nozzle enhance, overcoming flow resistance due to viscosity and ensuring uniform air entrainment and liquid injection. Consequently, at higher pressures, the outlet velocity distribution for fuels with different blending ratios converges. This indicates that at elevated pressures, viscosity’s impact on air entrainment and velocity distribution is reduced, improving fuel injection performance.

**Fig 10:**
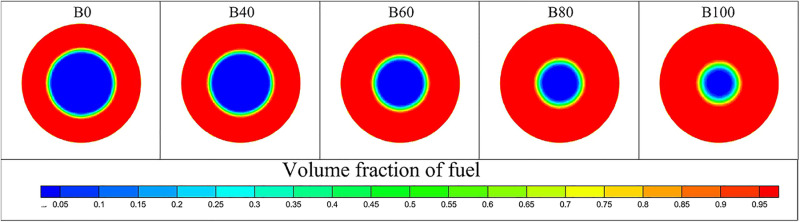
Liquid phase volume fraction distribution contour at nozzle outlet for aviation biofuels with different blending ratios.

Fig 10 shows the liquid-phase volume fraction distribution at the nozzle outlet for different aviation biofuel blending ratios at a fuel supply pressure of 0.2 MPa. Red areas represent fuel, and blue areas represent air. As the blending ratio increases, the liquid-phase volume at the nozzle outlet also increases, indicating that liquid film thickness grows with higher blending ratios.

**Fig 11:**
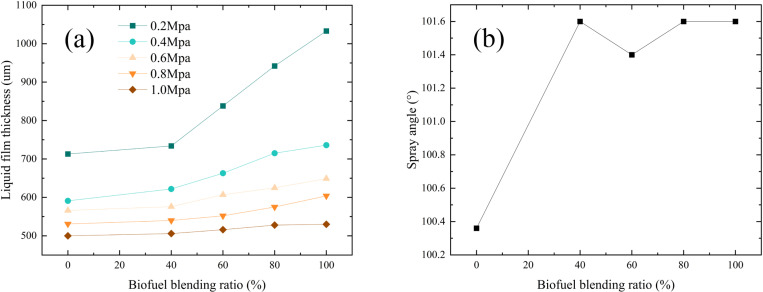
Variation of liquid film thickness and spray cone angle for aviation biofuels with different blending ratios. (a) Liquid film thickness, (b) Spray cone angle.

The liquid film thickness increases with the biofuel blending ratio, as shown in Fig 11. The smallest thickness is observed for pure conventional fuel (B0), while the largest is for pure biofuel (B100). This trend is attributed to the higher viscosity of biofuels, which thickens the liquid film. At an inlet pressure of 0.2 MPa, the thickness increases by 46.0% as the blending ratio rises from 0% to 100%. At 0.6 MPa, the increase is 12.9%, and at 1.0 MPa, it is only 4.2%. This indicates that at higher pressures, the influence of blending ratio on liquid film thickness diminishes. This is because, at higher pressures, the fuel atomizes more effectively, reducing the impact of the blending ratio on the liquid film. Additionally, the spray cone angle shows minimal variation across blending ratios, suggesting that the blending ratio has little effect on spray cone angle under the tested conditions.

These variations can be attributed to the influence of the blending ratio on the physical properties of the fuel, particularly changes in viscosity. Yao et al. [39] observed that higher viscosity reduces the fluidity of the liquid, making it more difficult for the liquid to form a thin film, thereby increasing the liquid film thickness. This effect is more pronounced at lower pressures, where the reduced kinetic energy and shear forces are insufficient to overcome the flow resistance caused by higher viscosity. However, at higher pressures, the kinetic energy and shear forces at the nozzle outlet increase significantly, allowing the liquid to break up and atomize more effectively. As a result, the influence of the blending ratio on liquid film thickness diminishes at elevated pressures.

This result aligns with the prediction of Equation (4), which states that as viscosity increases, the liquid film thickness also increases. However, an increase in pressure drop can mitigate the variation in liquid film thickness caused by changes in viscosity.

### 3.3 Effect of fuel temperature on atomization performance

To further investigate the effect of temperature on the atomization performance of aviation biofuels, a systematic study is conducted on fuels with different blending ratios (B0, B40, B60, B80, B100) under varying temperature conditions (0°C, 25°C, 50°C). The results show that, at a fuel supply pressure of 1.0 MPa, the trends in outlet velocity and liquid film thickness with temperature are generally consistent across all five fuels.

**Fig 12:**
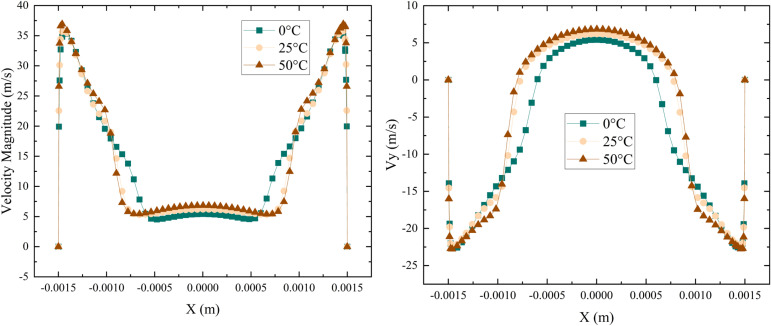
Velocity distribution at the outlet of aviation biofuels at different temperatures.

Fig 12 shows the radial distribution of axial and total velocity for B100 fuel at 0°C, 25°C, and 50°Cunder a 1.0 MPa fuel supply pressure. While elevated temperatures typically reduce viscosity and enhance injection in many fuels, these effects are especially pronounced in high-blend biofuel such as B100 due to its inherently higher viscosity relative to traditional jet fuel. As temperature increases, the viscosity drop in B100 is more substantial, leading to improved spray formation and atomization efficiency. This finding is supported by several studies in the literatures [40,41], which demonstrate that elevated temperatures improve injection velocity and atomization efficiency due to a reduction in fuel viscosity. Specifically, the reduction in viscosity with rising temperature facilitates better atomization of the fuel, thus improving both injection performance and combustion efficiency [42,43].

**Fig 13:**
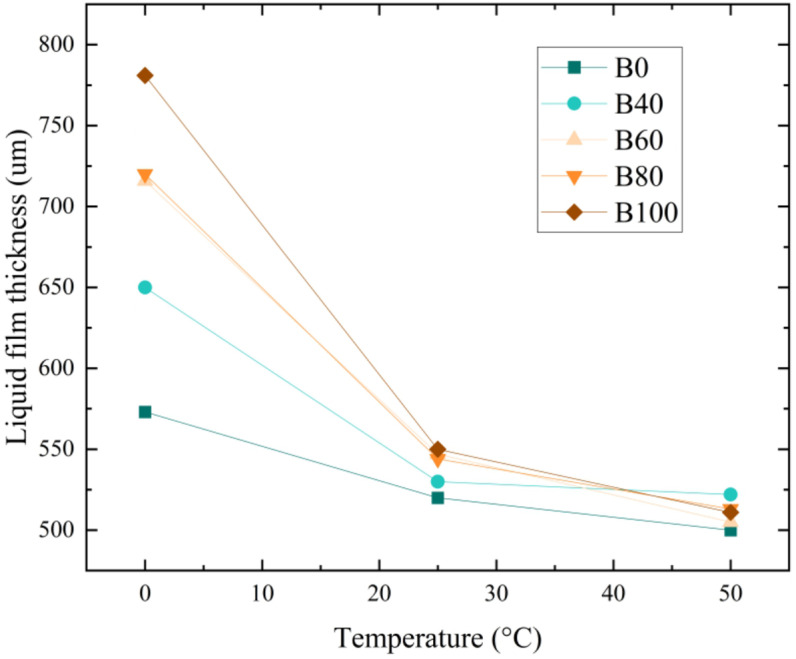
Variation in liquid film thickness of aviation biofuels at different temperatures.

Fig 13 illustrates the variation in liquid film thickness for aviation biofuels (B0, B40, B60, B80, and B100) at different temperatures under an inlet pressure of 1.0 MPa. The results show that as temperature increases, the liquid film thickness decreases for all fuels, with higher-blending-ratio fuels (such as B100) exhibiting the most significant reduction. The low-blending fuel (such as B0), the liquid film thickness decreased by 14.6%, whereas for the high-blending fuel (such as B100), it decreased by 52.8%. This phenomenon is primarily attributed to the substantial decrease in fuel viscosity at elevated temperatures, which enhances atomization performance.

**Fig 14:**
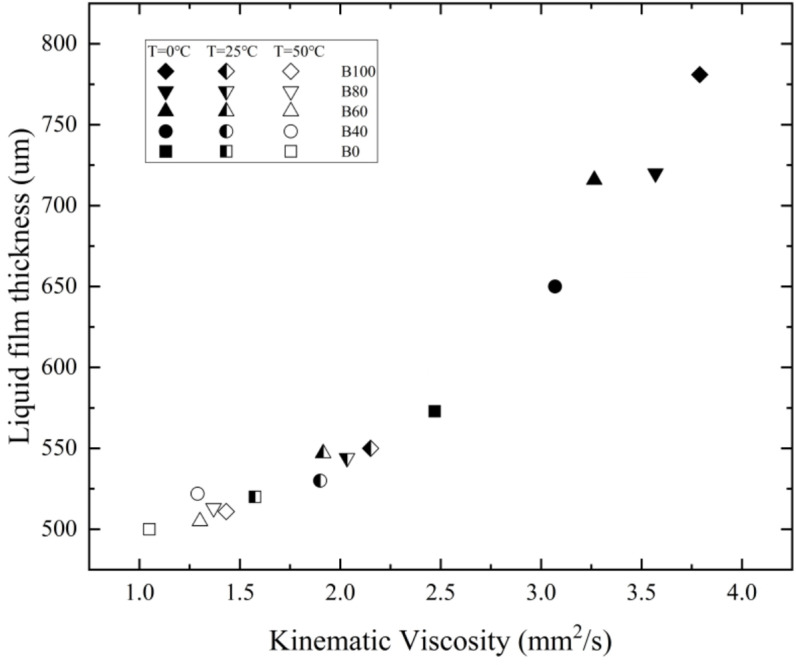
Effect of viscosity on liquid film thickness of aviation biofuels at different temperatures and blending ratios.

Fig 14 illustrates the relationship between the viscosity and liquid film thickness of aviation biofuels under different temperature and blending ratio conditions, with viscosity as the independent variable. From the figure, it is evident that there is a nonlinear positive correlation between viscosity and liquid film thickness, meaning that as viscosity increases, the liquid film thickness also increases. Specifically, when the viscosity is high, the response of the liquid film thickness to changes in viscosity is more sensitive, whereas at lower viscosity levels, the sensitivity of liquid film thickness to viscosity changes is weaker. This phenomenon indicates that the influence of viscosity on liquid film thickness varies across different viscosity ranges, and the dependence of liquid film thickness on viscosity increases with higher viscosity.

At 0°C, the fuel viscosity is relatively high, and the liquid film thickness is more sensitive to viscosity changes, with a considerable difference in viscosity between fuels of varying blending ratios, leading to significant variations in liquid film thickness. As the temperature rises to 50°C, fuel viscosity decreases, and the sensitivity of liquid film thickness to viscosity diminishes. At the same time, the viscosity difference between fuels of different blending ratios becomes smaller, resulting in reduced differences in liquid film thickness. At low temperatures, high-blending fuels exhibit significantly thicker liquid films compared to low-blending fuels; however, at higher temperatures, the difference between them becomes smaller. As a result, the liquid film thickness of high-blending fuels decreases more significantly with temperature.

## 4. Conclusions

This paper presents a simulation study using Fluent to investigate the atomization performance of aviation biofuels with different blending ratios in a centrifugal nozzle. The effects of nozzle inlet pressure, fuel temperature, and blending ratio on outlet velocity, liquid film thickness, and spray cone angle are analyzed in detail. The main conclusions are as follows:

1) The VOF method provides accurate simulations with less than 10% deviation from empirical data, confirming the reliability of the results.
2) Increased inlet pressure significantly improves atomization performance. Outlet velocity rose by 127.0%, while liquid film thickness decreases by 41.8%. The spray cone angle remains largely unchanged.
3) At low pressures, the blending ratio significantly impacts atomization performance. High-blending fuels produce liquid films up to 46% thicker than low-blending fuels and have lower outlet velocities. At 1.0 MPa, the difference in liquid film thickness narrows to 4.2%.
4) Fuel temperature significantly affects atomization performance, with marked differences between low- and high-blending fuels. As temperature increases from 0 to 50°C, outlet velocity rises while liquid film thickness decreases. The greater the blending ratio, the larger the decrease in liquid film thickness with temperature. For pure RP3, the decrease is 14.6%, while for pure biofuels, it is 52.8%. This shows that higher-blending fuels are more sensitive to temperature, with a larger reduction in liquid film thickness.
5) In actual engine use of high-blend aviation biofuels, it is recommended to increase the nozzle inlet pressure and fuel temperature to achieve atomization performance comparable to conventional fuels.

## 5. Future work

This study presents a numerical investigation of the atomization characteristics of high-blend aviation biofuels at pressures ranging from 0.2 MPa to 1.0 MPa and various temperatures. While this work provides valuable insights into the atomization performance of biofuels under these specific conditions, it does not address the atomization behavior under other critical operating conditions. Specifically, conditions such as cold-start and near-lean blow-out scenarios, which are also essential for assessing the practical applicability of high-blend biofuels in aviation engines, were not covered in this study. As a next step, future research will focus on conducting experimental studies to explore the atomization performance under these additional conditions, providing a more comprehensive understanding of biofuel behavior in real-world engine operations.

## Supporting information

S1 Data Data. (ZIP)

S1 File Figures. (ZIP)
