# Supplementary figures and images for: Numerical study on the atomization performance of aviation biofuel with high blending ratio
Source: PLoS One. 2025 May 6;20(5):e0321880. doi: 10.1371/journal.pone.0321880 (PMC12054894; doi:10.1371/journal.pone.0321880)

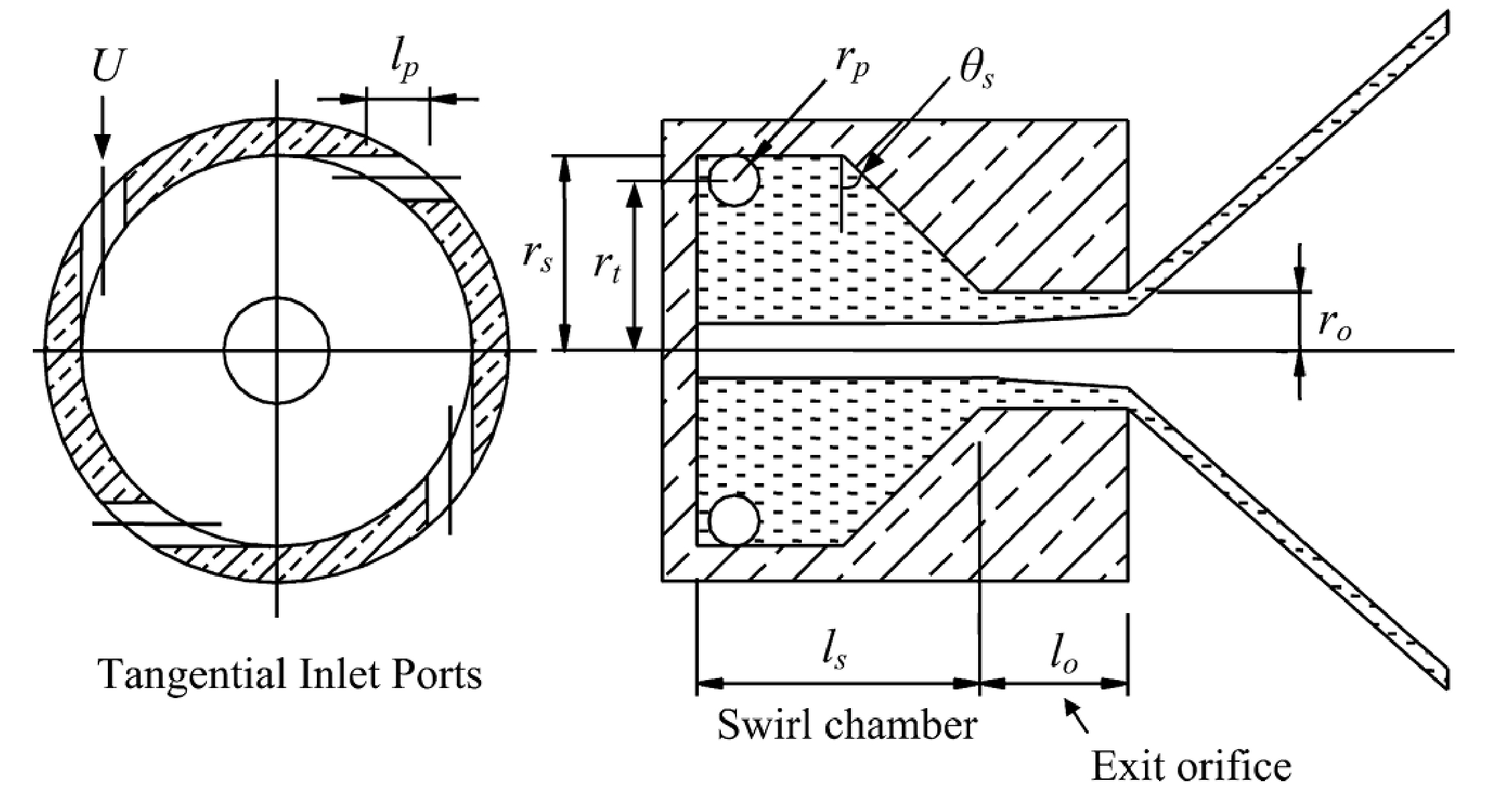

Supplement: S1 File — (ZIP) [file pone.0321880.s002.zip › Figures/Fig1.tif]

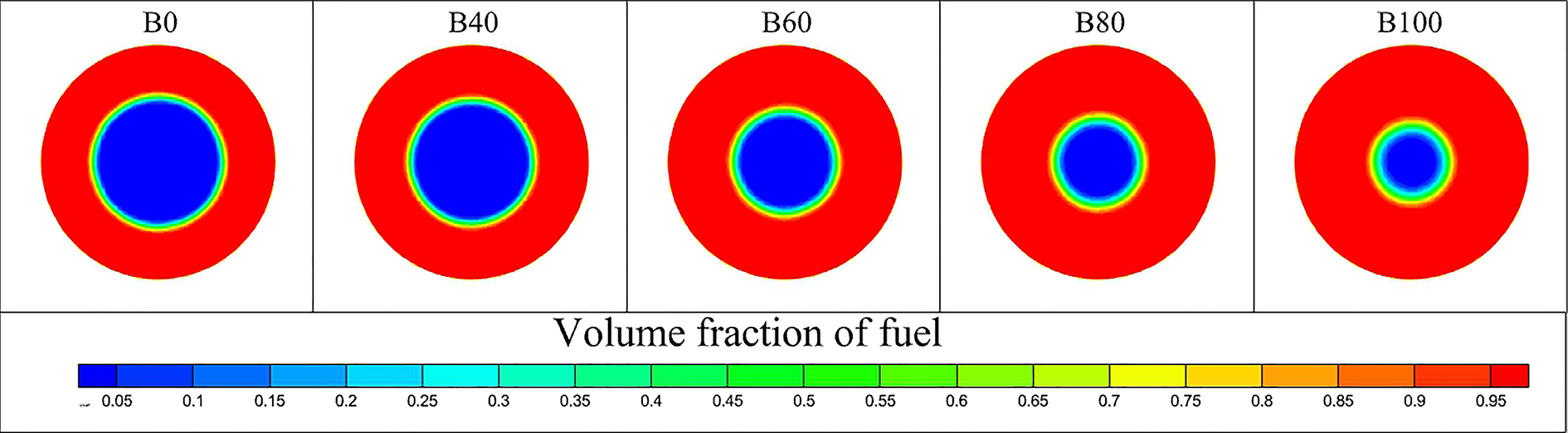

Supplement: S1 File — (ZIP) [file pone.0321880.s002.zip › Figures/Fig10.tif]

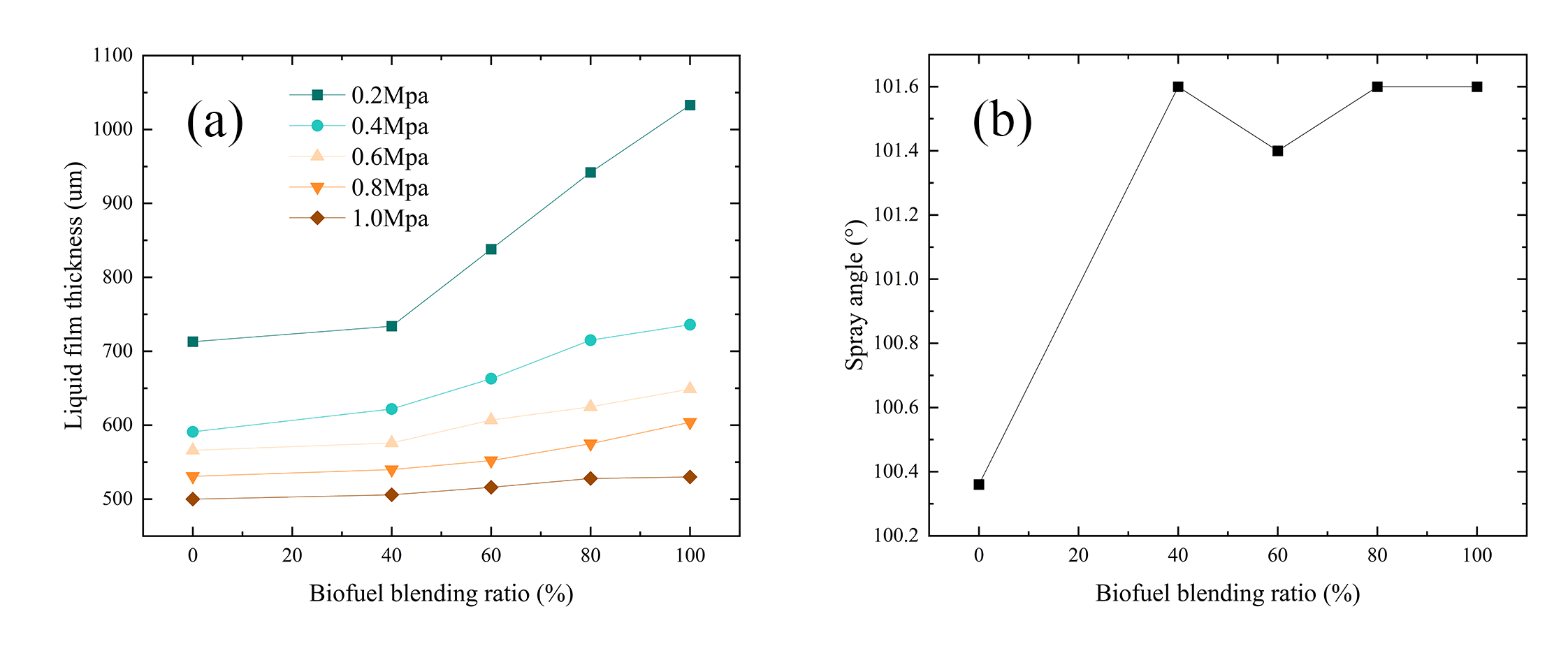

Supplement: S1 File — (ZIP) [file pone.0321880.s002.zip › Figures/Fig11.tif]

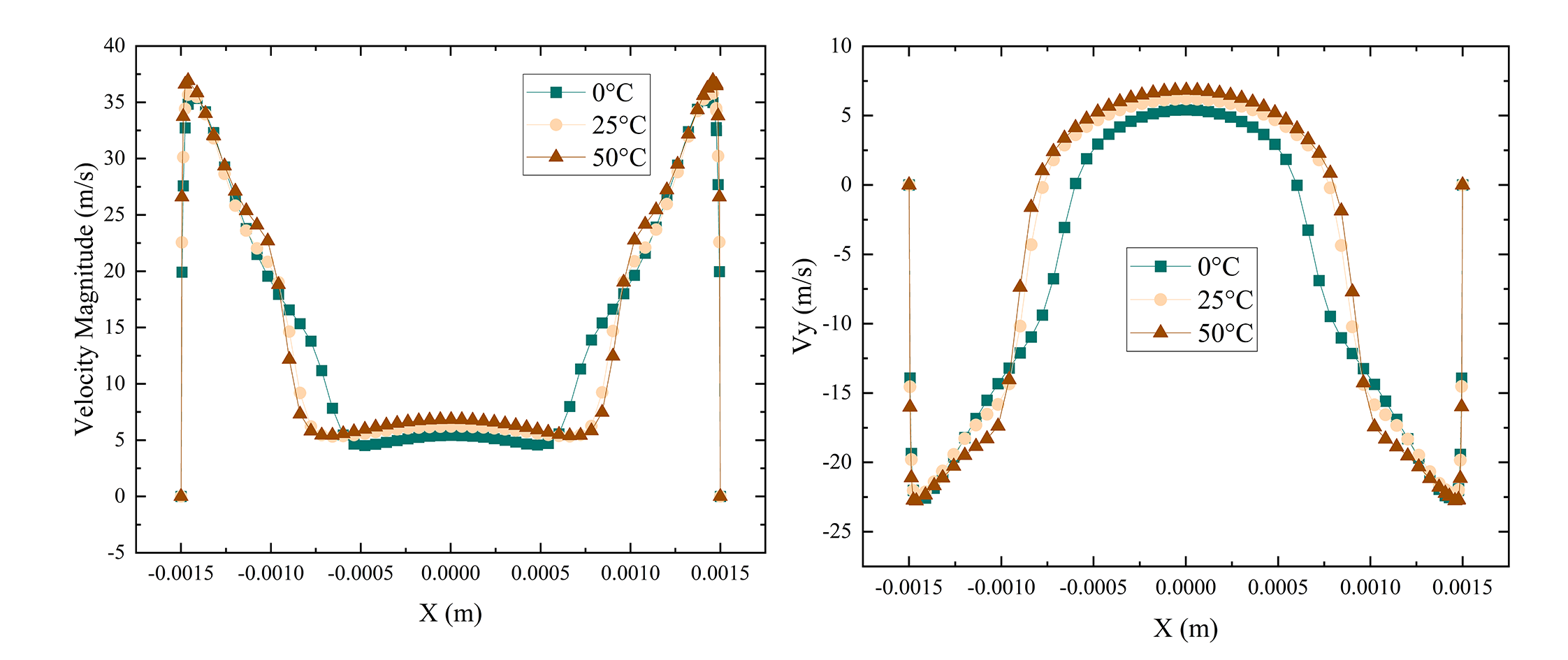

Supplement: S1 File — (ZIP) [file pone.0321880.s002.zip › Figures/Fig12.tif]

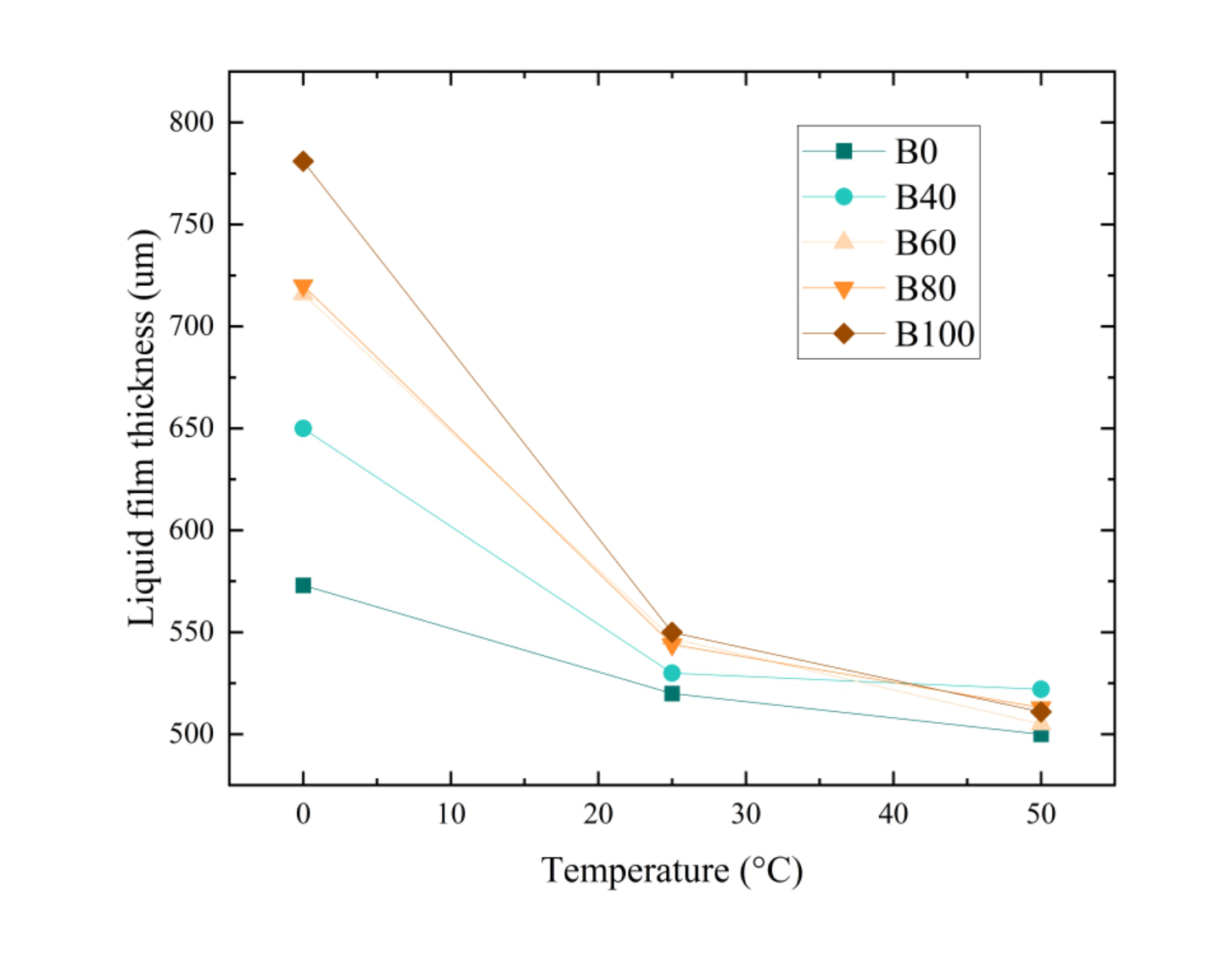

Supplement: S1 File — (ZIP) [file pone.0321880.s002.zip › Figures/Fig13.tif]

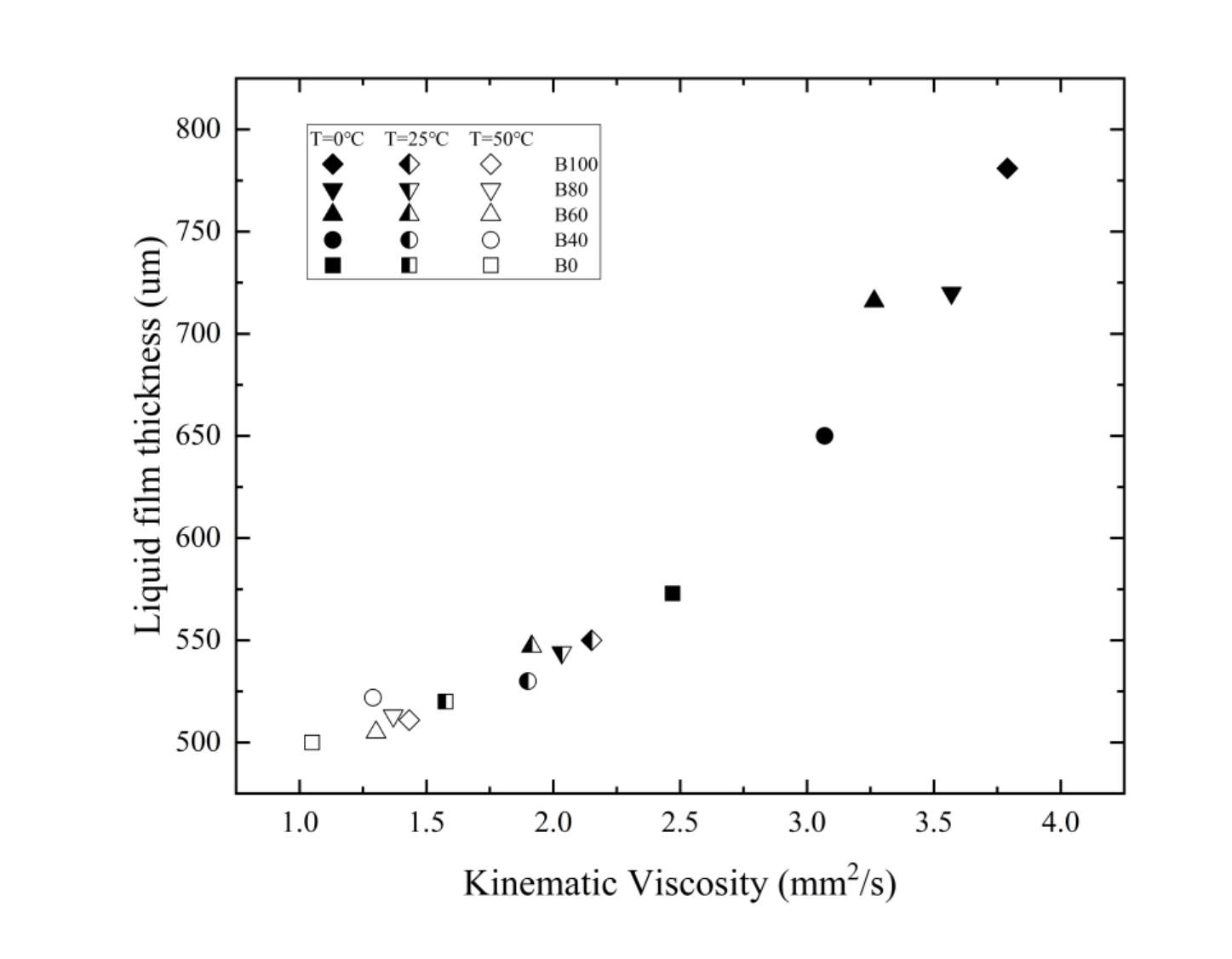

Supplement: S1 File — (ZIP) [file pone.0321880.s002.zip › Figures/Fig14.tif]

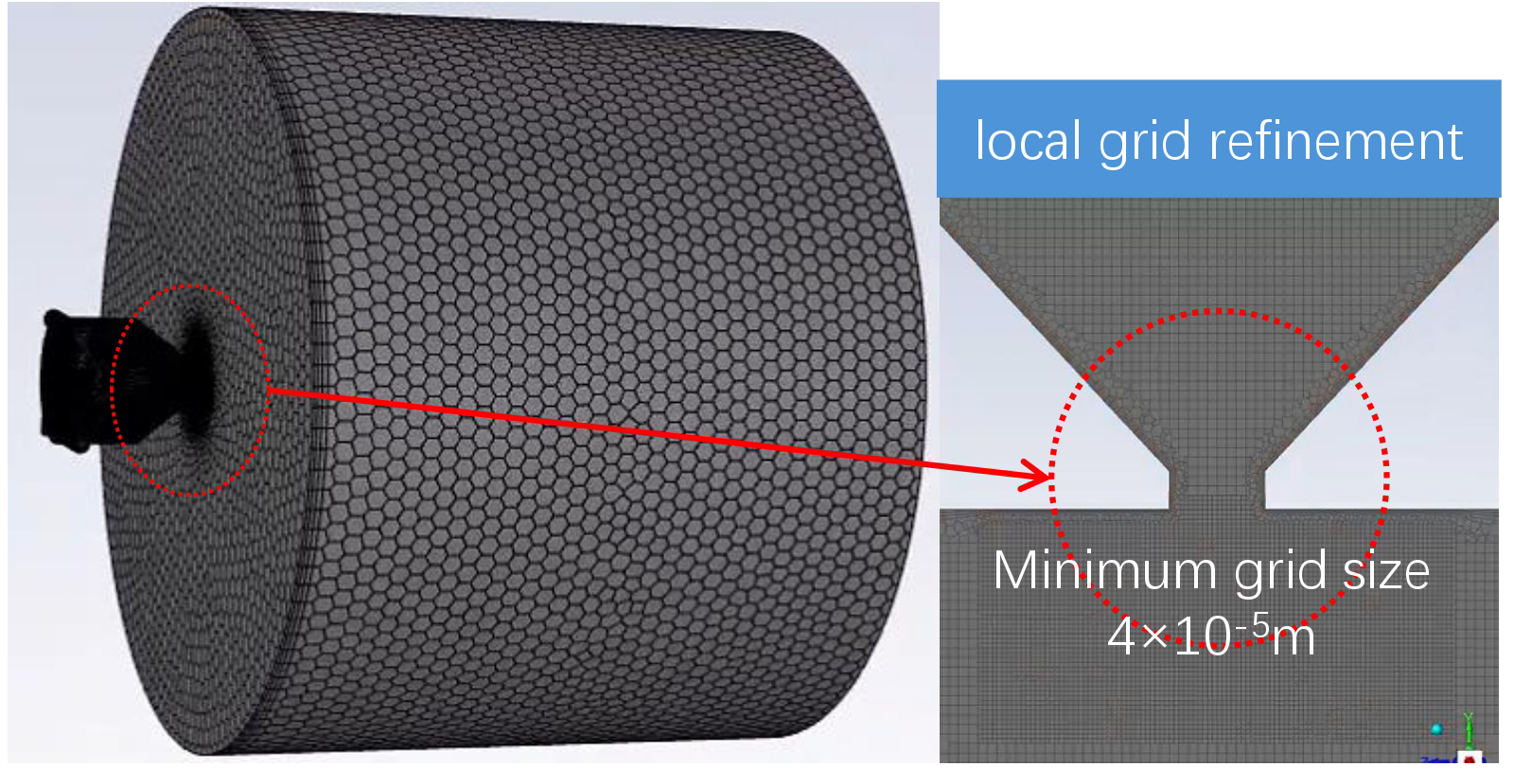

Supplement: S1 File — (ZIP) [file pone.0321880.s002.zip › Figures/Fig2.tif]

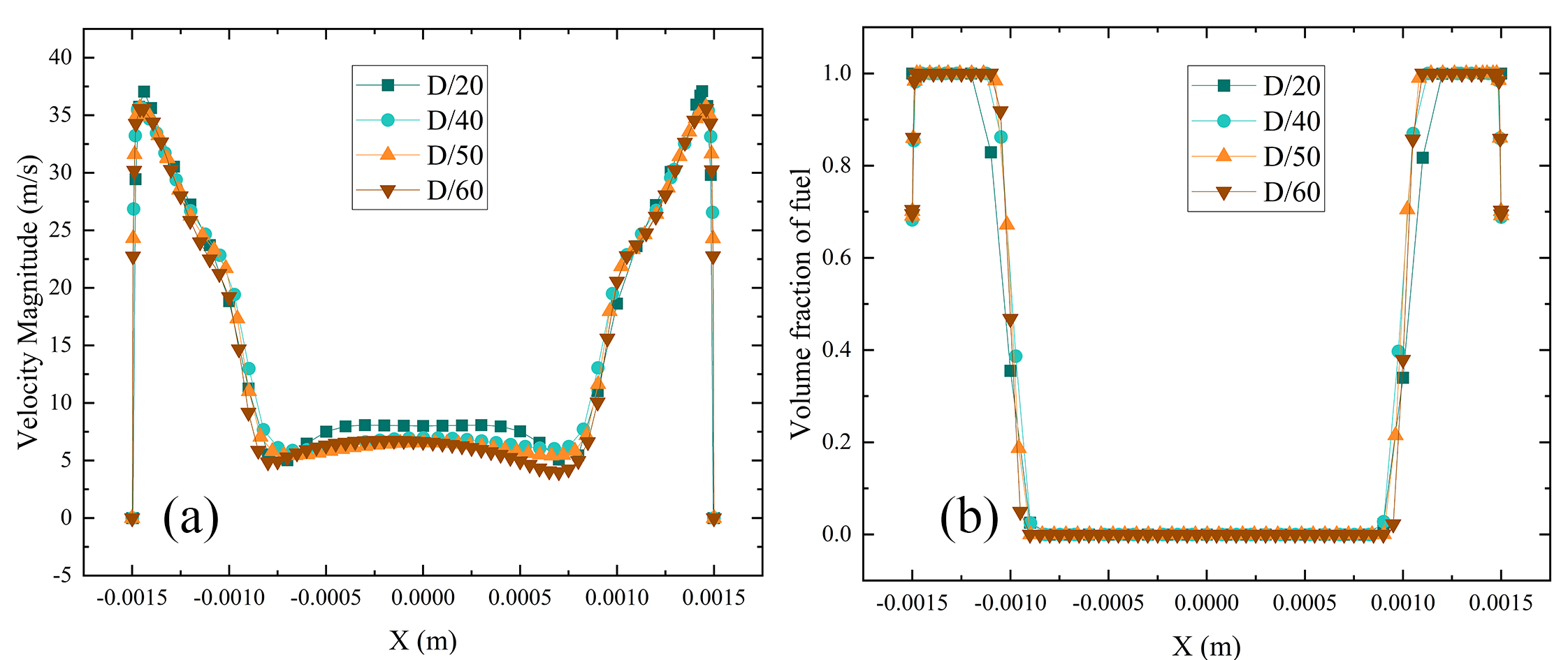

Supplement: S1 File — (ZIP) [file pone.0321880.s002.zip › Figures/Fig3.tif]

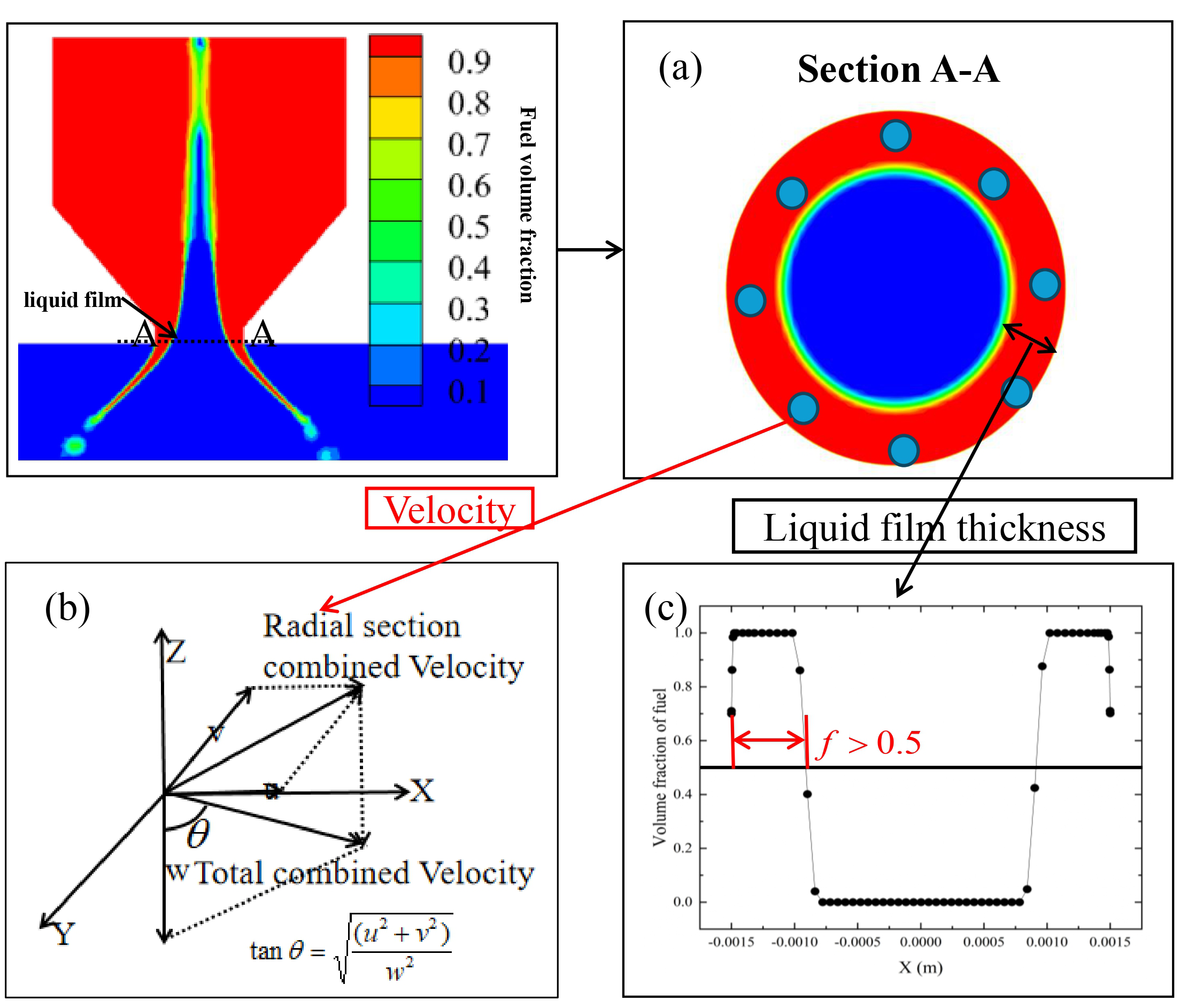

Supplement: S1 File — (ZIP) [file pone.0321880.s002.zip › Figures/Fig4.tif]

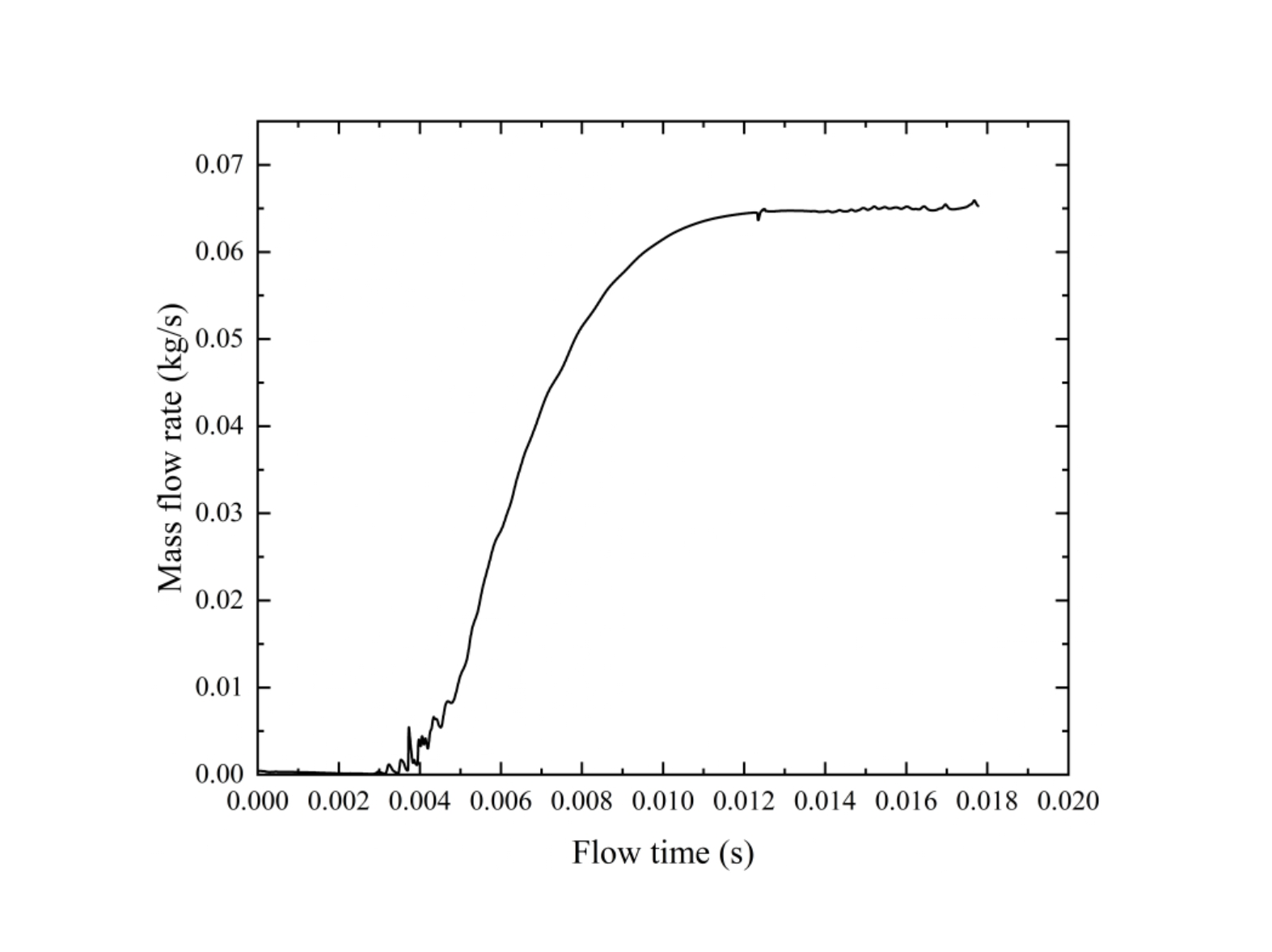

Supplement: S1 File — (ZIP) [file pone.0321880.s002.zip › Figures/Fig5.tif]

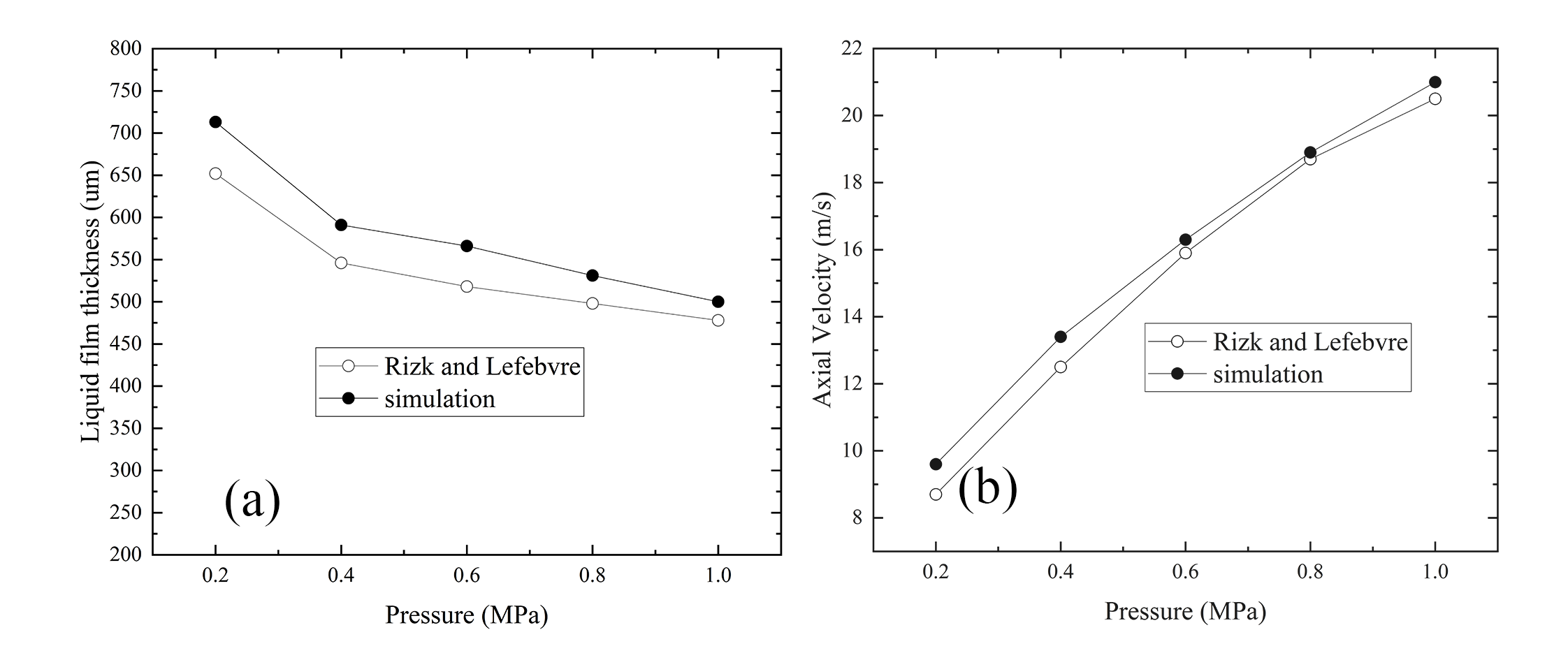

Supplement: S1 File — (ZIP) [file pone.0321880.s002.zip › Figures/Fig6.tif]

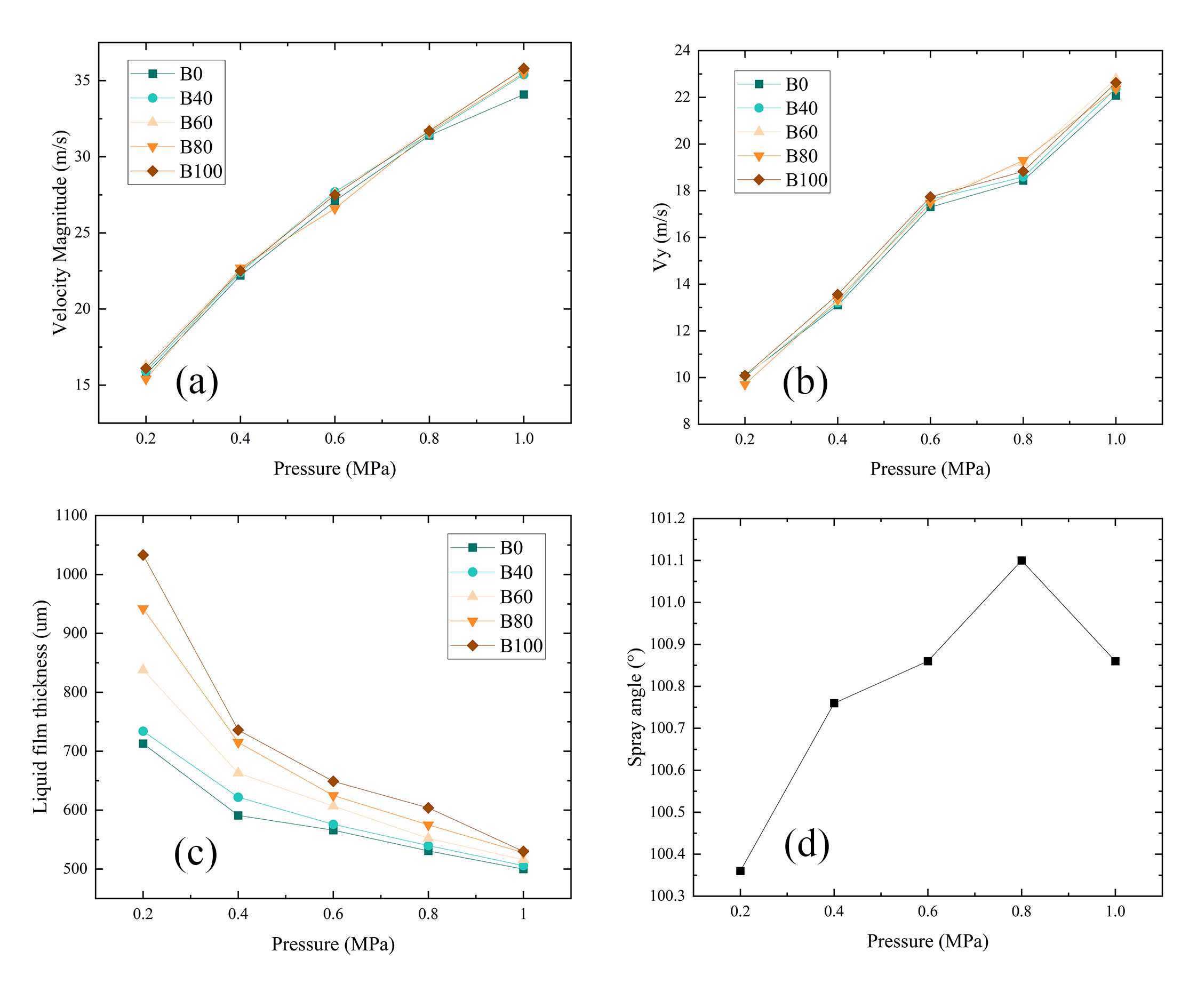

Supplement: S1 File — (ZIP) [file pone.0321880.s002.zip › Figures/Fig7.tif]

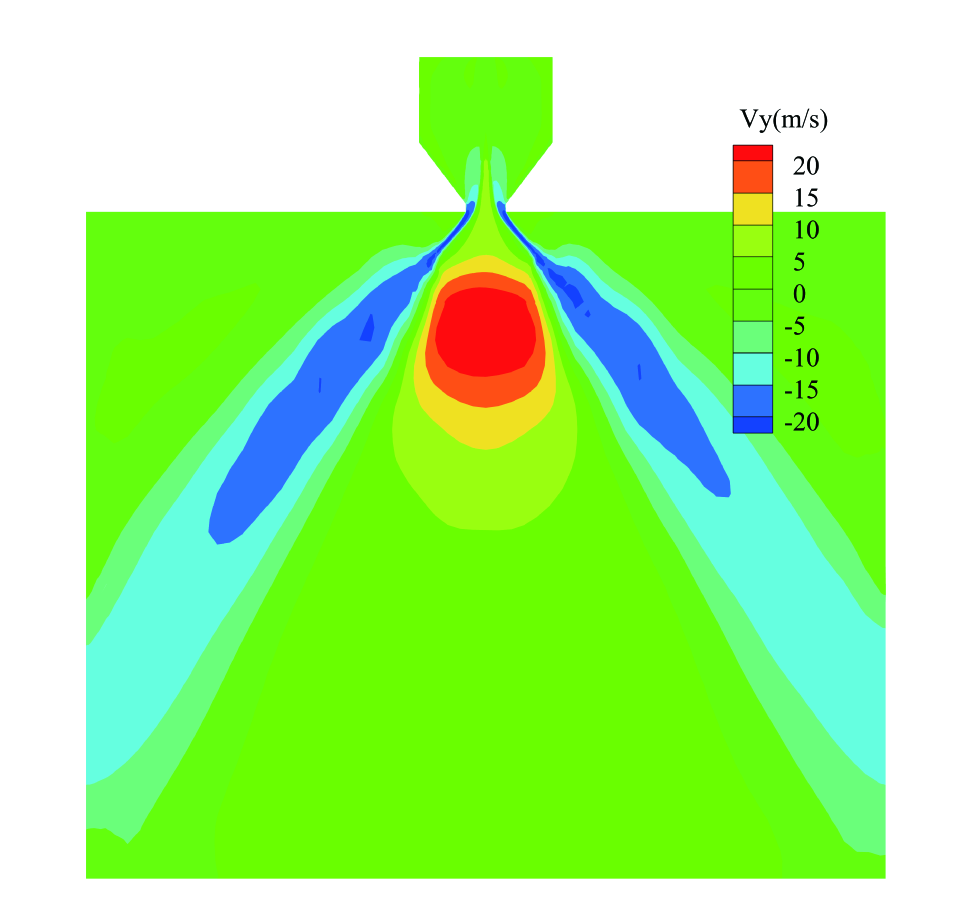

Supplement: S1 File — (ZIP) [file pone.0321880.s002.zip › Figures/Fig8.tif]

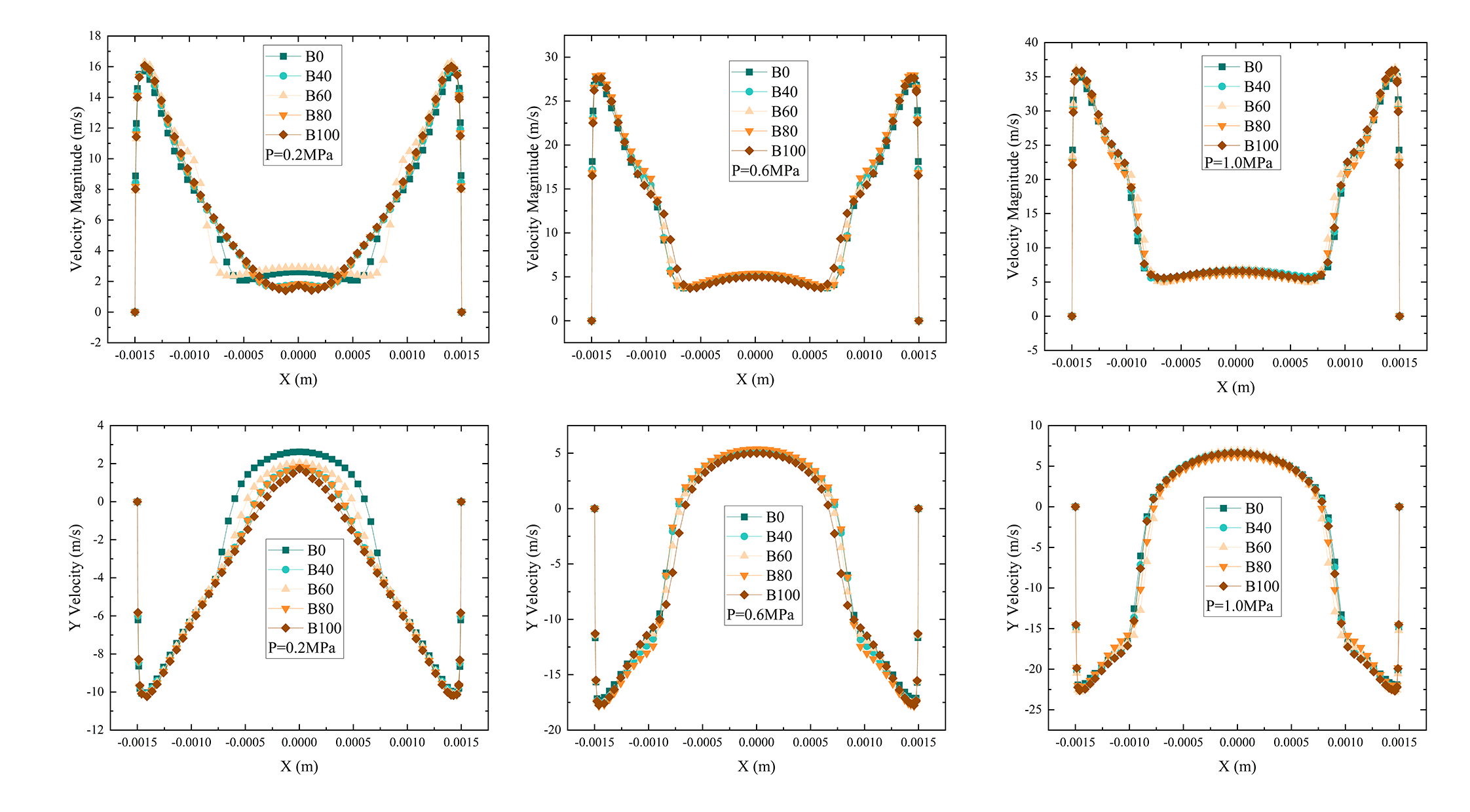

Supplement: S1 File — (ZIP) [file pone.0321880.s002.zip › Figures/Fig9.tif]
